# Regulation of Vascular Injury and Repair by P21-Activated Kinase 1 and P21-Activated Kinase 2: Therapeutic Potential and Challenges

**DOI:** 10.3390/biom14121596

**Published:** 2024-12-13

**Authors:** Chuting Han, Mengying Zhu, Yiting Liu, Yan Yang, Jun Cheng, Pengyun Li

**Affiliations:** Key Laboratory of Medical Electrophysiology of Ministry of Education and Medical Electrophysiological Key Lab of Sichuan Province, Institute of Cardiovascular Research, Southwest Medical University, Luzhou 646000, China; 20224099120006@stu.swmu.edu.cn (C.H.); 20234099120012@stu.swmu.edu.cn (M.Z.); 20234099120026@stu.swmu.edu.cn (Y.L.); wyangyan@swmu.edu.cn (Y.Y.); lzcj1221@swmu.edu.cn (J.C.)

**Keywords:** P21-activated kinase, vascular remodeling, angiogenesis, vascular permeability, inflammation, signaling pathway

## Abstract

The PAK (p21-activated kinases) family is a class of intracellular signal transduction protein kinases that regulate various cellular functions, mainly through their interactions with small GTP enzymes. PAK1 and PAK2 in the PAK kinase family are key signal transduction molecules that play important roles in various biological processes, including morphological changes, migration, proliferation, and apoptosis, and are involved in the progression of many diseases. Abnormal expression or dysregulation of PAK1 and PAK2 may be associated with several diseases, including cancer, neurological diseases, etc. The current research mainly focuses on studying the role of PAK and PAK inhibitors in the regulation of cancer progression, but relatively few reports are available that explore their potential role in cardiovascular diseases. Vascular injury and repair are complex processes involved in many cardiovascular conditions, including atherosclerosis, restenosis, and hypertension. Emerging research suggests that PAK1 and PAK2 have pivotal roles in vascular endothelial cell functions, including migration, proliferation, and angiogenesis. These kinases also modulate vascular smooth muscle relaxation, vascular permeability, and structural alterations, which are critical in the development of atherosclerosis and vascular inflammation. By targeting these activities, PAK proteins are essential for both normal vascular physiology and the pathogenesis of vascular diseases, highlighting their potential as therapeutic targets for vascular health. This review focuses on recent studies that offer experimental insights into the mechanisms by which PAK1 and PAK2 regulate the biological processes of vascular injury and repair and the therapeutic potential of the current existing PAK inhibitors in vascular-related diseases. The limitations of treatment with some PAK inhibitors and the ways that future development can overcome these challenges are also discussed.

## 1. Introduction

P21-activated kinases (PAKs) are a family of serine/threonine protein kinases; they are crucial for regulating diverse cellular processes in cardiovascular homeostasis and diseases [1,2]. PAKs consist of six members, PAKs 1-6. The overexpression or mutational activation of PAK isoforms frequently occurs in various human tumors [3]. While the PAK family members have been closely implicated in several aspects of cancer progression, such as proliferation, improved cell survival, and cell cycle progression [4], their specific role in cardiovascular diseases remains less elucidated. In recent years, increasing evidence suggests that PAK dysregulation is also involved in the regulation of endothelial cells (ECs) or vascular smooth muscle cells (VSMCs), making it important in the regulation of vascular homeostasis and remodeling [5,6]. Therefore, the malfunction of PAKs is associated with the development and progression of various cardiovascular diseases [2], further highlighting the significance of targeting PAKs as a promising therapeutic strategy in cardiovascular medicine. This review aims to elucidate recent advances in understanding the PAK1 and PAK2 signaling pathways and to provide a comprehensive overview of the roles of PAK1 and PAK2 in the regulation of vascular remodeling, angiogenesis, vascular permeability, inflammation, and atherosclerosis, all of which are crucial for the pathophysiological process of cardiovascular diseases. We also discuss the recent advances in the development of PAK1 and PAK2 inhibitors based on their structure types. Furthermore, we provide our perspective on future challenges of targeting these kinases in the pathogenesis of vascular diseases.

## 2. The Structure and Molecular Pathways of PAK1 and PAK2

### 2.1. Molecular Structure and Species Homology of PAK1 and PAK2

**Figure 1:**
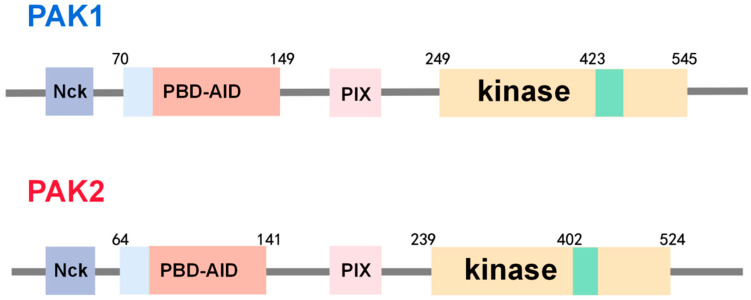
The schematic representation of the domain architecture of PAK1 and PAK2 in mammals. The Nck domain is shown in dark blue. The light blue area represents the self-inhibition dimer interface, which overlaps with both the p21-binding domain and the inhibition region shown in red. Another SH3-binding domain, PIX, is depicted in pink, while the yellow section represents the kinase domain. Finally, the activated dimer interface shown in green is situated in the kinase domain.

The PAK family comprises evolutionally conserved proteins expressed in various tissues [7]. Based on structural and functional similarities, they are classified into two groups: Group I (PAKs 1-3) and Group II (PAKs 4-6). The GTPase-binding domain (GBD) and the kinase domain of these two groups of PAKs share approximately 50% amino acid identity [8]. PAK1 and PAK2 are the most studied isoforms, with 91% sequence identity in their kinase domains [9,10]. Group I PAKs are composed of a regulatory domain (RD) in the N-terminal and a kinase domain (KD) in the C-terminal [11]. The N-terminus comprises a p21-binding domain (PBD) that partially overlaps with the auto-inhibitory domain (AID). Proline-enriched regions and exchange factor binding domains are also present in Group I PAKs [12]. PAKs were first identified as protein kinases that interact downstream of Rho GTPases, such as Cdc42 and Rac1 [7]. Upon binding with Cdc42/Rac1 at the Cdc42-binding domain, a conformational change occurs in the kinase region, leading to PAK activation [1]. Figure 1 depicts the domain structures of PAK1 and PAK2. PAK1 is widely expressed in various tissues, with particularly high levels in the brain, muscle, and spleen [13]. The percentage of homology to the human isoform is 99.26% in mice, 99.08% in rats, and 96.7% in dogs. While PAK2 is widely expressed, with particularly high expression in ECs [6], the percentage of homology to the human isoform is 97.14% in mice, 96.56% in rats, and 95.8% in dogs (the data were obtained from the UniProt).

### 2.2. The Activation Mechanism of PAK

**Figure 2:**
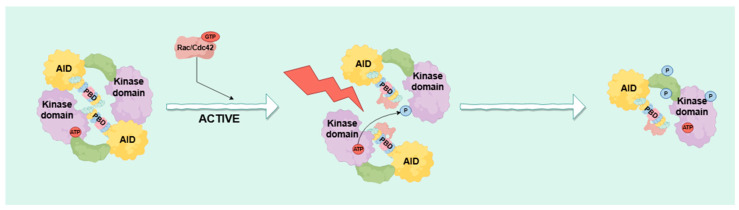
The activation process of PAK. PAK contains a PBD domain that can bind the active forms of Cdc42 and Rac 1 in the small GTPase family. When Cdc42 or Rac1 are in the active state, they bind to the PBD domain of PAK, causing a conformational change in PAK.

PAK demonstrates a range of activation mechanisms which are sensitive to a variety of external stimuli, such as signals originating from G protein-coupled receptors (GPCRs) and receptor tyrosine kinases (RTKs) [14]. As shown in Figure 2, PAK1 and PAK2 undergo multiple autophosphorylation upon interaction with appropriate activators, and small G proteins like Cdc42 and Rac can activate PAK1 through binding to its regulatory domain [15]. Intriguingly, PAK1 can also undergo activation independently of external signals through membrane localization [16]. Meanwhile, PAK2 activation is responsive to various stress factors, including high osmotic pressure, impaired DNA, sphingosine, ganglioside, phosphatidic acid, ionizing radiation, or serum deprivation [17,18,19]. Specific lipids can activate PAKs, but this mechanism differs from G protein-mediated activation, and elevated lipid levels can inhibit PAK kinase viability [20]. PAK2 is activated differently from PAK1, as it can undergo caspase-3-mediated restriction protease cleavage, leading to apoptosis-promoting fragments to regulate cellular activity under varying levels and types of stress [21]. PAK activation illuminates its diverse roles in the regulation of cellular functions and provides a foundation for potential therapeutic interventions in conditions linked to dysregulated PAK signaling [16].

### 2.3. The Substrates and Signal Pathways of PAK

P21-activated kinase (PAK) is crucial in regulating vital cellular processes like cell proliferation, migration, survival, and gene transcription [3]. PAK1 and PAK2 have numerous substrates, such as myosin light-chain kinase (MLCK), Bcl-2 family antagonist (BAD), Raf, mitogen-activated protein kinase (MAPK), extracellular signal-regulated kinase1 (MEK1), and LIM domain kinase (LIMK) [5,22]. PAK could regulate cytoskeletal-related, rearrangement-related factors, such as MLCK, LIMK, integrin-linked kinase (ILK), Merlin, Arpc1b, TCoB, and OP18 [7]. PAK1 also activates fine filament protein A [23], which is essential for cytoskeletal reorganization as it participates in growth factor-induced cofilin phosphorylation through LIM-kinase [24]. Moreover, PAK also stimulates multiple signaling proteins like JNK, β-catenin, and AKT/mTOR, to regulate cell survival and apoptosis [5]. Additionally, PAK influences transcription and translation by phosphorylating factors like PCBP1, histone H3, Aurora A, and polo-like kinase 1, as well as transcription factors like FKHR, CTBP1, and SNAI1 [16,25].

## 3. PAK1 and PAK2 Expression in Vascular Cells and Immune Cells

### 3.1. PAK1 and PAK2 Expression in the Vascular Cells

The blood vessel wall is composed of three main layers: tunica intima, tunica media, and tunica adventitia. PAK1 and PAK2 are expressed in all of the ECs located in the intima layer of the vessels [6,16]. PAK1 primarily regulates the morphology, migration, and proliferation of ECs, thereby affecting the vascular stability and the repair process [16]. Similarly, PAK2 is also implicated in these processes and plays a crucial role in modulating the function of ECs, contributing to angiogenesis [5,24].

PAK1 and PAK2 are also expressed in the SMCs within the media layer of the vasculature [16,26]. These kinases are involved in the regulation of the contraction, migration, and proliferation of the SMCs, which in turn affect the contractile function and structural remodeling of the vessel wall [27]. Their involvement is particularly important in pathological processes such as hypertension and atherosclerosis [28]. However, in the adventitia layer, the expression of PAK1 and PAK2 is rarely reported. The main cell types in adventitia are fibroblasts [29], macrophages, and other immune cells, which are involved in the structural support and functional regulation of blood vessels [30]. Therefore, further research is required to explore the expression and activity of PAK in different cell types within the vascular wall, which could provide valuable insights into vascular biology and potential therapeutic targets for vascular disorders.

### 3.2. Expression of PAK1 and PAK2 in Immune Cells

**Figure 3:**
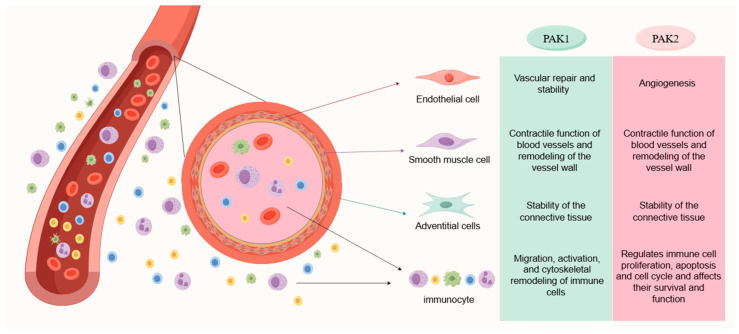
Roles of PAK1 and PAK2 in vascular cells. PAK1 primarily influences cytoskeletal dynamics and cell migration to regulate vascular stability and repair by targeting various cell types across the three tunics of blood vessels. PAK2, on the other hand, predominantly impacts angiogenesis, immune cell proliferation, and cell cycle progression. Collectively, both PAK1 and PAK2 have the capacity to modulate cell adhesion, vascular wall remodeling, and the stability of tissue connections.

Immune cells circulate within the blood vessels and are also capable of traversing the vessel walls to enter or exit the perivascular tissue [31]. The perivascular immune cells include macrophages, dendritic cells, lymphocytes, neutrophils, and mast cells, all of which collaborate to enhance immune surveillance and response [32]. PAK1 and PAK2 are the predominant PAK isoforms found in leukocytes [33]. PAK1 mainly regulates the migration, activation, and cytoskeletal remodeling, affecting the activation of T cells and the phagocytic function of macrophages [16]. PAK2, on the other hand, has been reported to play a crucial role in the polarization of macrophages [34], regulating the proliferation, apoptosis, and cell cycle [21]. The dysfunction in PAK1 and PAK2 is implicated in the regulation of vascular function and immune responses [5]. The different roles of PAK1 and PAK2 in various cell types are depicted in Figure 3.

## 4. PAK1 and PAK2 in the Regulation of Various Vascular Biological Functions

### 4.1. PAK1 and PAK2 in Vasoconstriction and Relaxation

Vasoconstriction and relaxation are important physiological processes that regulate the vasculature [35], and they play a key role in maintaining blood pressure, blood flow, and tissue blood supply [36]. Abnormal vasoconstriction or relaxation is closely associated with a variety of vascular diseases, especially hypertension, atherosclerosis, coronary heart disease, and heart failure [37]. The PAK family plays an important role in regulating vasoconstriction and vasodilation, mainly by affecting the function of SMCs, ECs, and macrophages. It has been documented that substrate stiffness regulates the distribution and activation of non-muscle myosin II (NMII) in site- and kinase-specific phosphorylation through the Rac1/PAK1/pS1916-NMIIA and PKCζ/pS1935-NMIII signaling pathways. As a key kinase in this signaling pathway, PAK1 may be closely related to the phosphorylation of myosin regulatory light chain (RLC), which affects the contractile function of smooth muscle, which in turn may affect the contractile state of vascular smooth muscle [38,39,40], which is crucial for blood pressure and blood flow regulation [41]. PAK also interacts with the Rho GTPase signaling pathway to regulate the cytoskeletal reorganization [42], further affecting the contraction and relaxation of vascular smooth muscle. PAK, in ECs, especially PAK2, regulates the light chain by mono-phosphorylating myosin II; it induces EC contraction to increase vascular permeability and may affect vasodilation function by regulating the production of nitric oxide (NO) [6].

### 4.2. PAK1 and PAK2 in Vascular Remodeling

Accumulating studies have indicated that PAK1 and PAK2 play important roles in transmitting cellular signals and regulating a range of cellular functions, such as cell proliferation, survival, and apoptosis, making them important therapeutic targets in various cardiovascular disorders [14,16,43]. Vascular remodeling refers to the structural modifications that occur in blood vessels, encompassing arteries, veins, and capillaries, in response to diverse physiological or pathological stimuli. This process encompasses changes in the size, shape, and composition of the vessels [44], alterations in the behavior of vascular smooth muscle and ECs, modifications to the extracellular matrix, inflammatory responses, and the process of angiogenesis [45]. Gaining insight into vascular remodeling is essential for managing cardiovascular disorders, as it directly impacts the function of vessels and exerts a profound influence on the therapeutic decisions [46].

#### 4.2.1. PAK1 in Vascular Remodeling

PAK1 is intricately involved in the complex processes of vascular remodeling and responds to various stimuli that contribute to changes in vascular structure and function [16]. It participates in the regulation of the proliferation and migration of VSMC, which are essential for cellular movement and morphological transformations [47]. PAK1 also influences matrix metalloproteinases (MMPs) [48], EC function [49,50], and inflammatory responses [51]—all of which are pivotal aspects contributing to vascular remodeling [52,53]. Activation of PAK1 is a critical early step in cell survival pathways and can be triggered by a variety of cellular signals, such as growth factors, cytokines, and cell adhesion molecules [16]. PI3K serves as an essential intermediate molecule in this cell survival signal pathway and activates PAK1 through its direct activator Rac [54,55]. Moreover, PAK1 can also activate the MAPK pathway, which regulates cell proliferation [7]. Recent studies have highlighted PAK1 as a regulator of apoptosis in VSMCs [56], where its persistent activation is associated with pathological vascular remodeling [47]. Additionally, PAK1 has also been reported to be activated by angiotensin II via the AT1 receptor, promoting vascular remodeling [43]; PAK1 knockdown inhibits VSMC migration stimulated by angiotensin II [47] and suppresses the proliferation of VSMCs triggered by the platelet-derived growth factor [57]. Therefore, the present studies indicate that PAK1 plays a crucial role in regulating cellular processes and represents a promising therapeutic target in vascular remodeling-related diseases.

#### 4.2.2. PAK2 in Vascular Remodeling

PAK2 is also emerging as a key participant in vascular remodeling, responding to diverse stimuli that contribute to alterations in vascular structure and function [58], while PAK2 has not been as extensively studied as PAK1. Like PAK1, PAK2 is involved in cytoskeletal dynamics, regulation of MMPs, integrin-mediated signaling, and the modulation of hypoxia-inducible factor-1 alpha (HIF-1α) [17], indicating its complex and multifaceted influence on the development of vascular remodeling. 

**Figure 4:**
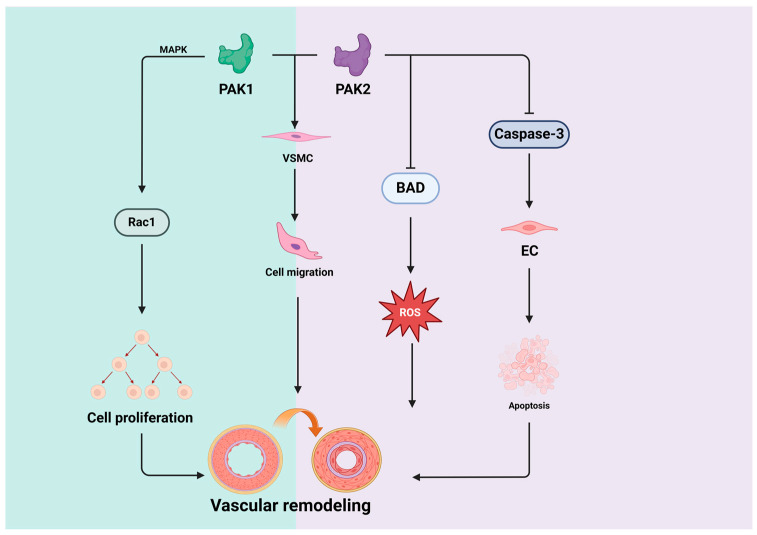
Roles of PAK1 and PAK2 in vascular remodeling. PAK1 exerts its influence on cell proliferation by activating Rac1 through the MAPK signaling pathway, while PAK2 mitigates ROS-induced activation and endothelial apoptosis by inhibiting BAD and Caspase-3. Collectively, PAK1 and PAK2 target VSMCs, impacting their migration and thereby playing a significant role in vascular remodeling.

However, many studies mainly focus on the regulatory role of PAK2 in vascular development (physiological remodeling) [6,59,60]. It has been well demonstrated that the expression of PAK2 in ECs plays a pivotal role in preserving embryonic viability and contributing to the formation of embryonic vasculature [6]. PAK2 is crucial in preserving genome integrity during the early embryonic stages, and any reduction in its activity leads to DNA damage, resulting in cell death through apoptosis or senescence [61]. The autophosphorylation of PAK2 activated by Cdc42 prevents the constitutive activation of caspase-3, rendering the cells resistant to apoptosis [21]. Moreover, the deficiency of PAK2 activity triggered the suppression of BAD phosphorylation, a crucial apoptotic precursor protein of the Bcl-2 family [62], leads to mitochondrial dysfunction and the subsequent release of cytochrome C and the formation of apoptotic complexes [63,64]. A significant increase in ROS levels in early embryos, which interferes with various biological processes [61], including the promotion of apoptosis and the aging of vascular ECs, results in angiogenic defects and early embryonic death [58].The above findings suggest that PAK2 is critical for maintaining embryonic viability and vessel formation, and its activity must be preserved for proper vascular development. Figure 4 depicts the detailed roles of PAK1 and PAK2 in vascular remodeling.

### 4.3. PAK1 and PAK2 in Angiogenesis

Angiogenesis is the process of forming new blood vessels from pre-existing capillaries or postcapillary veins [65]. This process involves the degradation of the vascular basement membrane; the activation, proliferation, and migration of ECs; and the reconstruction of new blood vessels and vascular networks [66]. Angiogenesis is crucial for a wide range of physiological processes, including wound healing and embryonic development [67].

#### 4.3.1. PAK1 in Angiogenesis

The proliferation and migration of ECs are essential processes for vascular repair [68]. PAK1 plays a pivotal role in orchestrating EC migration, which is a critical process in angiogenesis [16]. By influencing cytoskeletal dynamics and focal adhesion turnover, PAK1 modulates the cell cycle [16], fostering EC proliferation—a fundamental requirement for the expansive growth of blood vessel networks during angiogenesis [69]. Moreover, PAK1 actively contributes to the complex organization of ECs, facilitating the formation of tube-like structures, which is essential in the development of fully functional blood vessels [49]. It has been demonstrated that PAK1 can activate AKT, thereby promoting coronary artery angiogenesis [16]. This activation induces an increase in BAD phosphorylation, leading to the prevention of apoptosis [70]. Cdc42 serves as an upstream regulator of the PAK1 signaling pathway and mediates EC regeneration and vascular repair by activating the PAK1/AKT pathway [71]. Furthermore, PAK1 signaling was a key downstream mediator of Rac1 in angiogenesis [72,73]; PAK1 binds to Rac1 in a GTP-dependent manner and mediates vascular cell migration and angiogenesis [43,74]. Additionally, PAK1 plays a crucial role in cancer-related angiogenesis, proliferation, and vascular remodeling; the overexpression or nuclear translocation of activated PAK1 confers a proangiogenic function to mucinous fibrosarcoma, highlighting the vulnerable PAK1/STAT5B/CSF2 regulatory axis [5,16,25,75]. PAK1 influences transcription factors essential to angiogenesis, such as hypoxia-inducible factor 1-alpha (HIF-1ɑ), particularly under low-oxygen conditions [14,76]. Furthermore, PAK1 significantly modulates the activity of MMPs, playing a pivotal role in the dynamic remodeling of the extracellular matrix [16]. This regulatory mechanism is essential for facilitating the motility of ECs, which is a critical component of angiogenesis.

#### 4.3.2. PAK2 in Angiogenesis

PAK2 plays a pivotal role in the complex signaling pathways that are essential for angiogenic sprouting, serving as a bridge between focal adhesions and polarity signaling [77]. Paxillin, a structural component of focal adhesions [78], undergoes regulation through PAK-paxillin signaling, governing cell protrusion and migration [79]. In response to Angiotensin-1 (Ang-1), PAK2 facilitates paxillin reorganization within focal adhesions, triggering Cdc42 activation and initiating EC polarization, ultimately promoting angiogenic sprouting [17,77]. 

**Figure 5:**
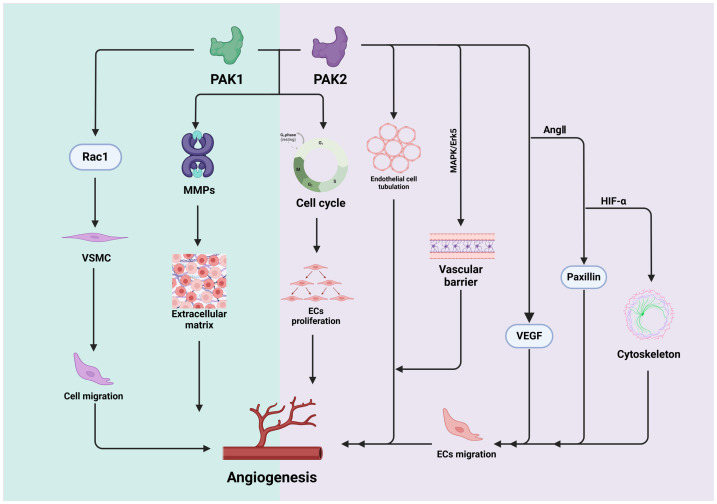
Role of PAK1 and PAK2 in angiogenesis. PAK1 and PAK2 can target MMPs and the cell cycle, thereby influencing the extracellular matrix and the proliferation of endothelial cells. Additionally, PAK1 can target Rac1, which affects the migration of VSMCs. PAK2, on the other hand, influences the formation of endothelial cells through the MAPK/Erk 5 pathway, as well as through the activation of VEGF and paxillin to regulate the cytoskeleton.

PAK2 is also crucial in tube formation, organizing ECs into tube-like structures while simultaneously regulating their proliferation and influencing the cell cycle, both of which are vital for the development of new blood vessels. As a vital mediator in EC function, PAK2 is instrumental in promoting both the developmental blood vessels and the maintenance of mature vascular homeostasis through Akt signaling [6]. Under physiological conditions, PAK2 enhances angiogenic factors, promoting EC attachment, migration, and cytoskeleton remodeling [59]. Through activating the MAPK/Erk5 pathway, PAK2 supports blood vessel formation and maintains the vascular barrier in adult animals [6]. The inhibition or dysregulation of PAK2-mediated signaling could suppress angiogenesis [80], suggesting its potential as a therapeutic target for diseases characterized by excessive blood vessel growth. However, comprehensive research is necessary to fully understand the intricate mechanisms of PAK2 in angiogenesis and to develop effective inhibitors for clinical applications. Figure 5 illustrates the roles of PAK1 and PAK2 in angiogenesis.

### 4.4. PAK1 and PAK2 in Vascular Integrity and Permeability

The structural integrity of ECs is crucial for maintaining the tight junctions and adhesion connections that partially regulate vascular permeability [81]. Disruption of these connections could lead to increased permeability, tissue edema, vascular rupture, and bleeding [82]. Several factors have been implicated in increased vascular endothelial permeability, including VEGF, oxidative stress, the renin-angiotensin system, oxidized LDL, homocysteine, and others [83]. The PAK kinase family regulates cytoskeleton-associated proteins and plays a crucial role in EC integrity and cell motility [25].

#### 4.4.1. PAK1 in Vascular Integrity and Permeability

PAK1 intricately regulates vascular permeability, a crucial factor in inflammatory responses and edema [2]. PAK1 is crucial for preserving EC integrity by affecting both tight and adherens junctions, which are essential for the proper functioning of the vascular barrier [84]. Additionally, PAK1 exerts a pivotal regulatory influence on cytoskeletal dynamics, impacting actin polymerization through its interaction with proteins such as myosin light chain (MLC) and LIM kinase [16]. These interactions are vital for maintaining the shape and integrity of cell junctions in EC [85]. Moreover, PAK1 could also regulate the phosphorylation and localization of occludin and claudins—proteins crucial for the formation and maintenance of tight junctions, thereby impacting vascular permeability [15,86].

Excessive PAK1 activity can increase the expression of VEGF, thus promoting angiogenesis [5,87], but VEGF secretion also induce PAK1 activation and contribute to increased vascular permeability [88], which is crucial in inflammation and angiogenesis [66,89]. Moreover, the overexpression of PAK1 could also affect intracellular junctions by adjusting E-cadherin distribution and inducing the phosphorylation of MLC, which reduces endothelial integrity and increases vascular permeability [84,90]. Additionally, by phosphorylating the substrate protein SHARP, PAK1 could modulate the Notch pathway, affecting EC apoptosis, endothelial integrity, and the expression of intercellular junction molecules [84,91,92]. These events collectively contribute to the complex processes that underlie vascular integrity [93]. Its multifaceted role highlights the critical importance of PAK1 in both the physiological and pathological aspects of vascular health.

#### 4.4.2. PAK2 in Vascular Integrity and Permeability

The role of PAK2 in the regulation of vascular permeability is complex and subject to numerous factors that have yielded conflicting results in previous studies. Various studies have suggested that PAK2 may impact barrier integrity in ECs, primarily by phosphorylating VE-cadherin at S665 [6], resulting in destabilized cell connections and increased vascular endothelial permeability [83]. Additionally, PAK2 has also been found to enhance endothelial permeability by mono-phosphorylating MLC at serine 19 [6], promoting cell contractility while simultaneously reducing cell contact, further contributing to increased permeability [94]. Specifically, the kinase activity of PAK2, in conjunction with its interaction with β-Pix, is critical for the formation of hematopoietic stem and progenitor cell filopodia, cytoskeletal integrity, and homing via Cdc42 [95]. PAK2 also indirectly controls cofilin activity through LIM kinase phosphorylation, which helps to regulate cytoskeleton remodeling and cell movement, leading to the formation of new adhesion points, thereby enhancing cell adhesion, motility, and increasing endothelial integrity [14,96]. Moreover, PAK2 functions as a cytoskeleton regulator [59,97], while Afadin (AF6) acts as a linker between intercellular adhesion molecules and the actin cytoskeleton, both of which play vital roles in maintaining vascular endothelial integrity [98]. Furthermore, the connection between PAK2 activation and the generation of reactive oxygen species (ROS) suggests a regulatory influence on EC function, which in turn contributes to changes in vascular permeability [99,100]. 

**Figure 6:**
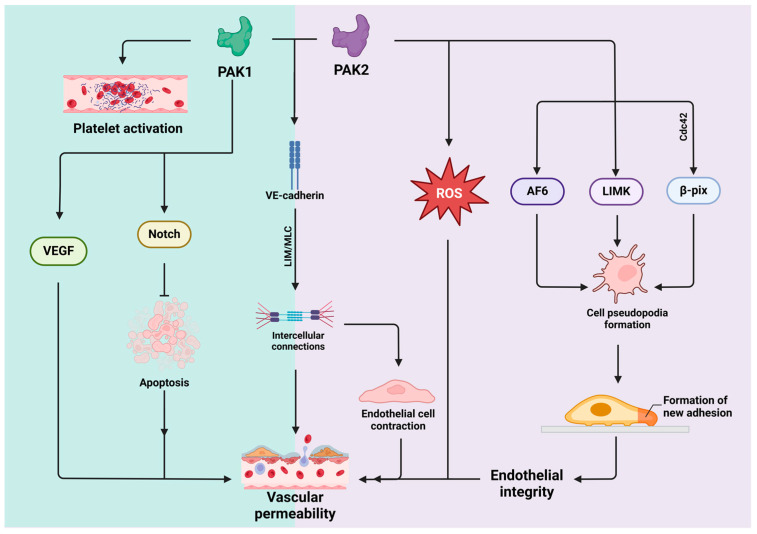
Roles of PAK1 and PAK2 in the regulation of vascular integrity and permeability. PAK1 and PAK2 collaboratively modulate vascular integrity and endothelial function by targeting VE-cadherin, which affects the stability of intercellular junctions. PAK1 exerts its individual influence on platelet activation and apoptosis through the Notch signaling pathway and directly impacts vascular permeability via VEGF. Meanwhile, PAK2 regulates the formation of cell pseudopodia and adhesion sites through interactions with AF6, LIMK, and β-Pix, thereby affecting endothelial permeability.

Contrary to previous findings, other studies have revealed that PAK2 may have a protective effect on the permeability of EC barriers [6,101,102]. This occurs through the stabilization of cellular connectivity, which promotes the maintenance of endothelial integrity, facilitates cytoskeletal rearrangement [103,104], and maintains optimal physiological function of the EC barrier [83]. For instance, the activation of Rac/PAK2 signaling in mice experiencing hypoxia has been demonstrated to decrease endothelial permeability [5]. Conversely, loss-of-function mutations in the PAK2 gene have been linked to cerebral hemorrhage and increased vascular permeability, albeit with no significant alterations in vascular patterns [105]. Moreover, in mature ECs, PAK2 deficiency has been found to promote cell apoptosis and acute defects in angiogenesis, culminating in increased permeability of the vascular endothelium [6]. The seemingly contradictory roles of PAK2 in the regulation of endothelial permeability may be attributed to its cell type-specific effects and the unique interplay between PAK1 and PAK2, which can lead to divergent cellular processes [15,58]. Furthermore, the impact of PAK2 on cellular events is highly dependent on the specific pathways involved [14,58]. Hence, extensive research is required to elucidate the complex mechanisms underlying the role of PAK2 in endothelial barrier regulation, which could significantly affect the development of novel therapeutic strategies. Figure 6 shows the roles of PAK1 and PAK2 in vascular integrity and permeability.

### 4.5. PAK1 and PAK2 in Vascular Inflammation Responses

Vascular inflammation is an immune reaction triggered by the vascular wall or adjacent tissues in reaction to factors such as injury, infection, or immune system activation [106]. The vascular inflammatory response can be localized or systemic, affecting the entire vasculature [45]. This can result in increased vascular permeability, thrombosis, or impaired vascular function, which is associated with a range of cardiovascular diseases [107]. PAK1 and PAK2 play significant roles in vascular inflammation by affecting EC activation, enhancing immune cell adhesion and migration, regulating cytoskeletal rearrangement, and modulating inflammatory signaling pathways [108].

#### 4.5.1. PAK1 in Vascular Inflammation Responses

PAK1 plays a crucial role in inflammatory signaling pathways, contributing to the development of various vascular diseases [16]. PAK1 could activate leukocytes, including neutrophils and macrophages, by regulating cytoskeletal dynamics, which are essential for cell polarization, adhesion, and migration towards inflammatory sites [14,109]. The activation of PAK1 also leads to the generation of ROS [110], a common feature in inflammatory responses, influencing antimicrobial activity and inflammatory signaling [111]. PAK1 is tightly involved in inflammatory pathways, activating NF-κB and driving the production of cytokines, chemokines, and adhesion molecules, thereby playing a key role in the inflammatory response [75]. Additionally, it regulates the MAPK signaling pathways, including ERK, JNK, and p38 MAPK, which are essential for conveying inflammatory signals from the cell membrane to the nucleus [112,113]. PAK1 also influences the activation of ECs during inflammation, affecting the expression of adhesion molecules and altering vascular permeability [16]. The dysregulation of PAK1 is associated with inflammatory conditions such as rheumatoid arthritis and inflammatory bowel disease, potentially fueling chronic inflammation through its abnormal activity [114]. Since the inflammatory response plays an important role in vascular diseases like atherosclerosis, aneurysm, etc. [115], understanding PAK1’s role is crucial for managing pathological inflammation and mitigating vascular damage.

#### 4.5.2. PAK2 in Vascular Inflammation Responses

**Figure 7:**
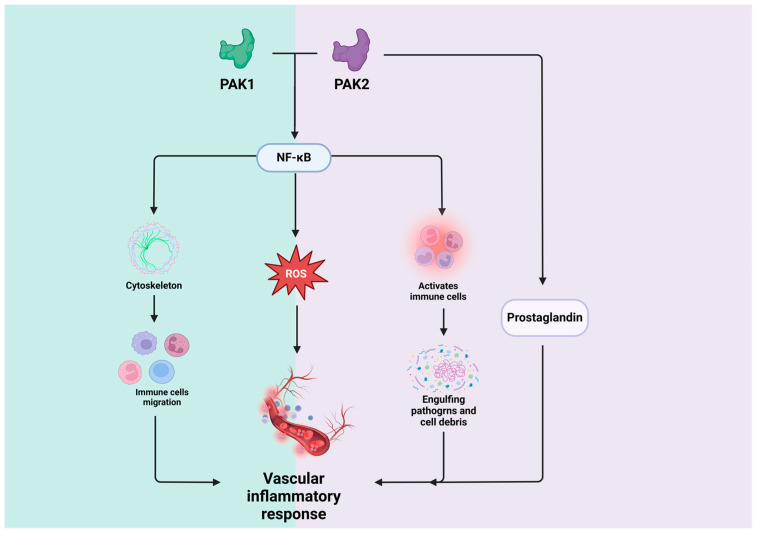
Roles of PAK1 and PAK2 in vascular inflammatory response. PAK1 and PAK2 can affect ROS and the activation and migration of immune cells through the NF-κB pathway, thus affecting the vascular inflammatory response; in addition, PAK2 can also act on prostaglandins alone to affect the vascular inflammatory response.

PAK2, like PAK1, has also been implicated in inflammatory signaling pathways [17] and plays a multifaceted role in the inflammation [2]. It is crucial in activating immune cells like lymphocytes and macrophages and modulating signaling pathways that govern immune cell responsiveness to inflammatory signals [21]. PAK2 plays a crucial role in regulating the dynamics of the cytoskeleton in immune cells, with a particular impact on actin polymerization, which is crucial for their migration and the formation of immunological synapses during immune responses [109]. Additionally, PAK2 is intricately involved in directing immune cell chemotaxis and migration toward inflammatory sites, influencing the cytoskeletal rearrangements that are crucial for cell movement [15]. It also modulates inflammation-related signaling, including pathways triggered by cytokines and chemokines, and enhances NF-κB activation [116]. Moreover, the activation of PAK2 is associated with the generation of ROS, contributing to the oxidative burst during immune responses [59]. PAK2 also controls inflammatory mediators such as cytokines and prostaglandins, amplifying the inflammatory response [117], and enhances the phagocytic capabilities of immune cells, reinforcing their role in immune defense [118]. The dysregulation of PAK2 has also been implicated in inflammatory diseases, such as atherosclerosis, rheumatoid arthritis, and diabetic vasculopathy, in which altered PAK2 activity may lead to chronic inflammation in perivessels [17]. The multifaceted role of PAK2 in the inflammatory response has a potential benefit for vascular disease. Figure 7 shows the roles of PAK1 and PAK2 in the vascular inflammatory response.

## 5. PAK Inhibitors and Potential Application in Treating Vascular Disorders

PAK inhibitors have been developed and can be divided into two categories depending on the binding sites within the PAK molecules, ATP-competitive inhibitors, and allosteric PAK inhibitors [119]. ATP-competitive PAK inhibitors, such as FRAX597, FRAX486, and FRAX-1036 [11,86], block PAK phosphorylation by targeting the ATP-binding pocket within the kinase domain and have higher inhibitory activity on PAK1-3 [120], whereas allosteric PAK inhibitors are a class of compounds that bind to a site on the PAKs other than ATP-binding site, inducing conformational changes that result in the inhibition of kinase activity. This kind of inhibitor, such as IPA-3 [121], has the potential to be more selective and to display discriminative inhibitory activity among PAK family proteins, as it takes advantage of the unique structural features of the target kinases [119].

PAK inhibitors have the advantage of targeting multiple signaling pathways in the pathogenesis of cardiovascular diseases [3]. However, the current research and the development of inhibitors for the clinical applications of PAK1 and PAK2 in the vascular field are relatively limited, and most studies have focused on their role in cancer and neurological diseases [16]. Theoretically, the inhibitors of PAK1 and PAK2 may also affect vascular function and related diseases [16]; modulating PAK1 and PAK2 activity will be useful in treating dysfunctional vascular problems, such as hypertension, diabetic retinopathy, and cerebrovascular diseases [17]. However, the specific application still needs further research and clinical verification.

There are relatively few studies on the specific applications of PAK1 and PAK2 agonists, as most studies have focused on their inhibitors [120,122,123,124,125,126,127,128]. The inhibitors and agonists of PAK1 and PAK2 are shown in Table 1. However, there are some preliminary studies suggesting that activating PAK1 and PAK2 may have some influence on certain vascular-related physiological processes [17], although the studies are still in an early stage. Furthermore, the poor druggability and selectivity limit their further development in preclinical/clinical trials [129]. Therefore, studies may focus on exploring the more specific PAK1 or PAK2 modulators to prevent or treat vascular pathologies. Understanding the molecular mechanisms underlying PAK-mediated vascular dysfunction could lead to the identification of novel therapeutic targets and the development of innovative strategies for the management of vascular diseases.

## 6. Conclusions and Perspectives

In recent years, an increasing number of studies have demonstrated PAK family kinases as significant players in both physiological and pathological processes, particularly highlighting their roles in cancer and cardiovascular disease. The existing literature indicates that PAK1 and PAK2 serve as pivotal transducers in the signaling pathways involved in the process of vascular injury and repair, thereby regulating multiple biological responses, such as cytoskeletal dynamics, cell adhesion, migration, and proliferation, affecting angiogenesis, vascular remodeling and permeability. In particular, PAK1 has also been implicated in the regulation of cell metabolism, oxidative stress, and inflammatory responses, which could have implications for cardiovascular health [14,140]. However, the pathophysiological mechanisms by which PAK1 and PAK2 influence vascular diseases are complex and not yet fully elucidated.

Understanding the role of PAK1 and PAK2 in the pathogenesis of cardiovascular diseases is crucial for developing targeted therapies. Currently, most studies of PAK inhibitors focus on cancer therapy, such as blocking tumor migration and angiogenesis. Despite the potential role of the PAK family members in the regulation of vascular function and their importance as therapeutic targets in diseases such as atherosclerosis, hypertension, and angiogenesis, the data on the clinical trials of PAK inhibitors are relatively limited. Future studies should investigate the expression and activity of PAK1 and PAK2 in the samples from human diseases and correlate these findings with clinical outcomes. Due to the complexity of the PAK signaling, the potential non-specificity of PAK inhibitors, and the heterogeneous expression in different cells within vessels, more preclinical studies are needed for the application of PAK inhibitors in vascular diseases to evaluate its safety and efficacy in regulating vascular function and treating vascular diseases.

In conclusion, an increasing number of studies suggest that PAK1 and PAK2 have emerged as key regulators of the various pathophysiological processes involved in vascular dysfunction. PAK1 and PAK2 inhibitors may become new treatments in the field of vascular diseases in the future. The development of inhibitors that are both potent and specific, with a favorable safety profile, is essential for clinical translation.

## Figures and Tables

**Table 1:** The main small molecule inhibitors and agonists of PAK1 and PAK2.

**Inhibitor**	**Structure**	**Classification**	**IC50**	**References**
G-5555	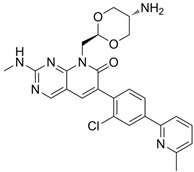	ATP-competitive inhibitors	PAK1 3.7 nM PAK2 50 nM	[130]
FRAX1036	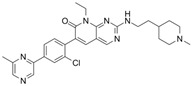	Selective depressant	PAK1 23.3 nM(Ki) PAK2 72.4 nM(Ki)	[131]
FRAX486	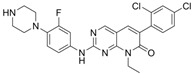	Selective depressant ATP-competitive inhibitors	PAK1 14 nM PAK2 33 nM	[125]
2,2′-Dihydroxy-6,6′-dinaphthyl disulfide	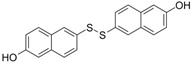	Nonspecific inhibitor	-	[132]
G-9791	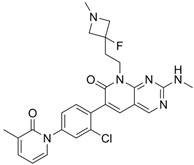	Selective depressant	PAK1 0.95 nM (Ki) PAK2 2.0 nM (Ki)	[133]
IPA-3	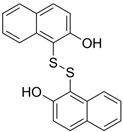	Non-ATP-competitive, Allosteric inhibitors	PAK1 2.5 μM	[134]
FRAX597	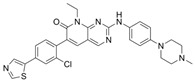	Selective depressant ATP-competitive inhibitors	PAK1 8 nM PAK2 13 nM	[120,134]
ZINC194100678	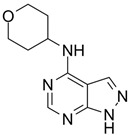	Small molecule inhibitors	PAK1 8370 nM	[135]
NVS-PAK1-1	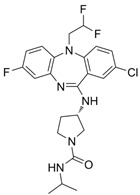	Allosteric inhibitor	PAK1 5 nM	[122]
NVS-PAK1-C	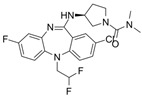	ATP-competitive, Allosteric inhibitor	PAK1 5 nM PAK2 270 nM	-
AZ13705339	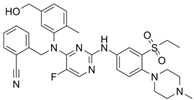	Selective depressant	PAK1 0.33 nM pPAK1 59 nM	[136]
PAK1-IN-1	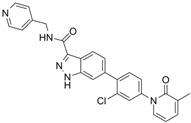	Selective depressant	PAK1 9.8 nM	*-*
ZMF-10	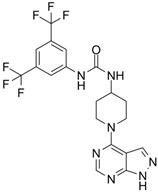	Selective depressant	PAK1 174 nM PAK2 1038 nM	[135]
AK963/40708899	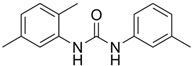	Selective depressant	PAK1 40 nM (IC50)	[137]
K-252a	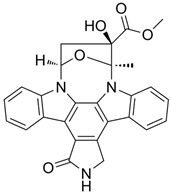	ATP-competitive inhibitors	PAK1 2.4 nM	[138]
Staurosporine	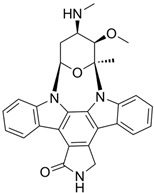	ATP-competitive inhibitors	PAK1 0.75 nM	[16,139]
**Agonists**	**Structure**	**Classification**	**IC50**	**References**
Fingolimod (FTY720)	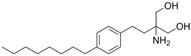	*-*	0.033 nM	[43]
